# Neutrophil NET Formation with Microbial Stimuli Requires Late Stage NADPH Oxidase Activity

**DOI:** 10.3390/antiox10111791

**Published:** 2021-11-09

**Authors:** Heather A. Parker, Harry M. Jones, Christopher D. Kaldor, Mark B. Hampton, Christine C. Winterbourn

**Affiliations:** Centre for Free Radical Research, Department of Pathology and Biomedical Science, University of Otago Christchurch, Christchurch 8011, New Zealand; heather.parker@otago.ac.nz (H.A.P.); harrymjones077@gmail.com (H.M.J.); cdavkaldor@gmail.com (C.D.K.); mark.hampton@otago.ac.nz (M.B.H.)

**Keywords:** neutrophil extracellular traps, NADPH oxidase inhibition, myeloperoxidase, hydrogen peroxide, microbial stimuli of NETs

## Abstract

Neutrophils respond to a range of stimuli by releasing extracellular traps (NETs), a mesh consisting of chromatin plus granule and cytoplasmic proteins. We have investigated NET release in response to phorbol myristate acetate (PMA), *Pseudomonas aeruginosa* (PAO1), *Staphylococcus aureus* and *Candida albicans*, and the involvement of NADPH oxidase (NOX2) and myeloperoxidase (MPO) activities. An oxidative mechanism was involved with each stimulus, and the NOX2 inhibitor diphenylene iodonium (DPI) gave almost total inhibition. Notably, DPI added up to 60–90 min after stimulation still gave significant inhibition of subsequent NET formation. As most of the NOX2 activity had already occurred by that time, this indicates a requirement for late-stage low-level oxidant production. Inhibition of histone citrullination did not suppress NET formation, indicating that this was not the essential oxidant-dependent step. With PMA and *P. aeruginosa* PAO1, MPO activity played an important role in the induction of NETs and MPO inhibitors added up to 30–90 min after stimulation suppressed NET formation. NET formation with *S. aureus* and *C. albicans* was insensitive to MPO inhibition. Thus, MPO products are important with some stimuli but not others. Our results extend earlier observations with PMA and show that induction of NETs by microbial stimuli requires late stage oxidant production. Others have shown that NET formation involves NOX2-dependent elastase release from granules. As this is an early event, we conclude from our results that there is more than one oxidant-dependent step.

## 1. Introduction

Release of neutrophil extracellular traps (NETs) is a well-established part of the neutrophil response in infection and inflammation [1,2,3]. Although NETs probably play a minor role in clearing micro-organisms relative to phagocytosis and intracellular killing [4], they are being increasingly recognized as contributors to the pathology of a number of inflammatory conditions including autoimmune disease, thrombosis, respiratory distress syndromes and cancer [1,2,3,5]. NETs are induced by a variety of agents, including the non-physiological but frequently employed protein kinase C activator phorbol myristate acetate (PMA), bacterial phagocytosis, ionomycin, cholesterol and urate crystals and various inflammatory mediators [1,2,6]. With some stimuli including LPS-activated platelets, NET formation can be a rapid, vital process [3,7]. However, with most stimuli, it is a slower, lytic process in which NET release occurs 2–4 h after stimulation. The latter mechanism commonly requires NADPH oxidase (NOX2) activity and generation of reactive oxygen species: NET formation is not seen when NOX2 is lacking, as in chronic granulomatous disease [8,9] or inhibited with diphenylene iodonium (DPI) [10,11].

Activation of NOX2 leads to the consumption of oxygen and production of superoxide radicals. These rapidly dismutate to hydrogen peroxide (H_2_O_2_), which is converted by the enzyme myeloperoxidase (MPO) to the highly bactericidal oxidant hypochlorous acid (HOCl). Other secondary derivatives can also be produced [12]. Although an oxidant requirement was recognized in early studies [8,9], how oxidants are involved in NET formation, or what species are important, is not well understood. Exogenous addition of specific oxidants or oxidant products has been used to try to identify what species are involved, but activation tends to require high, supraphysiological amounts [8,13,14] that could be acting simply as lytic agents [4]. Therefore, results cannot necessarily be extrapolated to the situation with endogenous oxidant production. However, partial inhibition by catalase when it is internalized by conjugation to polyethylene glycol [10] implies a role for H_2_O_2_. This is consistent with the proposal of Björnsdottir et al. [15,16] that the relevant oxidants are likely to be produced in low amounts at localized intracellular sites.

There is good evidence that MPO activity contributes to NET formation with some stimuli. This is strongest for PMA, in which case deficiency or inhibition of MPO suppresses or delays NET formation [10,17,18]. The MPO product, HOCl, has been shown to produce NETs [19,20], but high concentrations were used, and it is difficult to exclude a lytic response. However, decreased NET formation when chloride is limiting [19], and the ability of HOCl-derived chlorofatty acids to induce NETs [21] are consistent with a role for HOCl. On this basis, it is often assumed that NOX-dependent NET formation universally requires MPO. However, our previous finding that several microbial stimuli produce NETs that are sensitive to NOX2 inhibition but not to MPO deficiency or inhibition [10,13] implies that this is not universal.

The timing of oxidant involvement in NET formation is also not well understood. PMA and phagocytosis induce rapid NOX2 activation in neutrophils. Release of granule elastase into the cytoplasm has been proposed as an essential step in NET formation. It has been shown to be NOX-dependent and occur within an hour of PMA stimulation [18]. However, NET formation induced by PMA was inhibited by DPI added well after the addition of the stimulus [15]. This implies that oxidants produced during the initial oxidative burst are not sufficient to induce NETs. It is generally accepted that results obtained with PMA need verification with more physiological stimuli before extrapolating to a wider context [1,22]. Therefore, we have induced NET formation with *Candida albicans*, *Pseudomonas aeruginosa* PAO1 and *Staphylococcus aureus*, as well as PMA, to determine at what stage NOX2 activity is required, and whether or not MPO activity is involved. To do this, we have used DPI as a well-established inhibitor of NOX2 that blocks neutrophil superoxide production [12], DPI was added at various times after each stimulus and the potent MPO inhibitors, thioxanthine 1 (TX1) [23] and AZM198 [15], were used to examine the role of MPO.

## 2. Materials and Methods

### 2.1. Materials

Endotoxin-free water, Ficoll-Paque PLUS and an Enhanced Chemiluminescence (ECL, St. Paul, MN, USA) Plus Western blotting detection system were from GE Healthcare (Buckinghamshire, UK). Dextran was from MP Biomedicals (Santa Ana, CA, USA). RPMI 1640 without phenol red and fetal bovine serum (FBS) were from Gibco (Waltham, MA, USA). Columbia sheep blood agar plates were from Fort Richard Laboratories (Auckland, New Zealand), LB Miller from Fisher Bioreagents (Fisher Scientific, Hampton, UK) and Tryptone soya broth from Oxoid (Basingstoke, UK). Bacto peptone and yeast extract were from Becton Dickinson (Le Pont De Claix, France). Sytox green was obtained from Molecular Probes (Eugene, OR, USA).

Rabbit polyclonal anti-neutrophil elastase (Ab 21,595), rabbit polyclonal anti-histone H3 (citrulline R2 + R8 + R17; Ab 5103) and goat anti-rabbit dylight 594 (Ab 96,901) were from Abcam (Cambridge, UK). Fluoromount G was from eBioscience (San Diego, CA, USA). Paraformaldehyde was from ProSciTech (Kirwan, QLD, Australia). The MPO inhibitors TX1 and AMZ198 were a gift from AstraZeneca (Gothenburg, Sweden). Cl-amidine and ionomycin were from Calbiochem (San Diego, CA, USA). H_2_O_2_ (30% solution) was purchased from LabServ (Victoria, Australia). Nonidet P-40 was from Pierce Biotechnology (Waltham, MA, USA). Phosphate-buffered saline (PBS), Hank’s solution (HBSS), cOmplete™ protease inhibitor cocktail and all other chemicals and solutions were from Sigma-Aldrich (St. Louis, MO, USA).

### 2.2. Neutrophil Isolation

Neutrophils were obtained with informed consent from the peripheral blood of healthy volunteers under ethical approval from the Southern Health & Disability Ethics Committee, New Zealand (Wellington, New Zealand [URA/06/12/083]). Neutrophils were isolated by dextran sedimentation followed by Ficoll density centrifugation and removal of contaminating RBCs by hypotonic lysis [24]. They were suspended in RPMI (without phenol red) containing 10 mM HEPES and 2% heat inactivated FBS. Purity was assessed by flow cytometry and was routinely ≥ 98%. Autologous serum was obtained from whole blood collected without anticoagulant and was used immediately. For experiments with *C. albicans*, pooled human serum was heat inactivated (56 °C, 30 min) and stored at −20 °C.

### 2.3. Bacteria and Yeast Strains and Culture

*P. aeruginosa* PAO1 (ATCC 47,058) was purchased from American Type Culture Collection (ATCC) Australia. *S. aureus* 502A (27,217; ATCC) was obtained from the NZ communicable Disease Centre (Porirua, New Zealand). *C. albicans* was a clinical isolate. *P. aeruginosa* and *S. aureus* were grown in LB and tryptic soy broth, respectively. Bacteria and yeast were routinely cultured on sheep blood agar or yeast peptone dextrose (YPD) agar, respectively. For experiments, bacteria were cultured overnight at 37 °C with shaking, washed and suspended into HBSS, then centrifuged at 150× *g* for 5 min to remove clumped bacteria. *C. albicans* was cultured overnight in YPD at 30 °C (195 rpm), washed twice in PBS, then suspended in YPD. Bacterial and yeast number were calculated by measuring absorbance at 550 nm or 600 nm, respectively, and relating this to a previously determined standard curve based on colony counts. Prior to use, bacteria were opsonized in 10% autologous serum in HBSS (20 min, 37 °C, 6 rpm). Yeast was opsonized in 10% heat inactivated pooled human serum in RPMI (30 min, 37 °C, 6 rpm). Conversion of *C. albicans* to the hyphal form began during opsonization.

### 2.4. NET Induction

Neutrophils (1 × 10^6^/mL) were warmed to 37 °C and then stimulated with PMA (20 nM), *P. aeruginosa* PAO1 (MOI 10), *S. aureus* (MOI 10) or *C. albicans* (MOI 2). When required, diphenylene iodonium (DPI), TX1, AZM198 or rotenone (all at a final concentration of 10 µM) were added 10 min before stimulation or at the specified time points after stimulation. Cl-amidine (200 µM) was pre-incubated with neutrophils for 30 min prior to stimulation.

### 2.5. NET Quantification

Neutrophils (1 × 10^6^/mL) were transferred to black 96-well plates (100 µL/well) and incubated in the presence or absence of stimulant for up to 4 h at 37 °C in 5% CO_2_. The presence of NETs was assessed by staining with Sytox green (3 µM, 5 min). Fluorescence was measured using a POLARstar fluorescence plate reader (BMG Technologies, Hamburg, Germany) (excitation 485 nm, emission 520 nm). The fluorescence of unstimulated cells was subtracted from that of stimulated cells.

### 2.6. Immunofluorescence Microscopy

For detection of NETs, neutrophils (5 × 10^5^) were stimulated and seeded onto sterile glass coverslips in 24-well plates and incubated for 4 h at 37 °C in 5% CO_2_. After centrifugation (100× *g*, 5 min), the cells were fixed by adding an equal volume of 8% paraformaldehyde for 20 min at room temperature and permeabilized with 0.5% Triton X-100 (10 min). Samples were washed in PBS-tween20 (PBST) (0.05%) and then blocked overnight in 10% BSA at 4 °C. Samples were incubated with or without anti- neutrophil elastase antibody diluted 1/500 in 3% BSA for 1 h at 37 °C. Subsequently, samples were washed with PBST and incubated with goat anti-rabbit Dylight secondary diluted 1/5000 in 3% BSA for 1 h at 37 °C. Samples were washed with PBST followed by PBS, stained with Sytox green (200 nM) for 5 min, rinsed in water and mounted with Fluoromount G. Cells were viewed using a Zeiss Axio Imager fluorescence microscope (Jena, Germany). Images were captured with a Photometrics KAF1400 charge-coupled device camera and Zen Blue software (Zeiss, Oberkochen, Germany). For detection of citrullinated histone 3 (cit-H3), treated samples were treated with anti-cit H3 (1/500) instead of anti-elastase and processed as above.

### 2.7. Electron Microscopy

Neutrophils (2.5 × 10^5^) were stimulated with PMA (20 nM) with or without DPI (10 µM), seeded onto sterile glass coverslips in 24-well plates and incubated up to 240 min at 37 °C in 5% CO_2_. At intervals, cells were fixed at room temperature in 0.1 M cacodylate buffer (pH 7.5) containing 2% paraformaldehyde, 2.5% glutaraldehyde and 5% sucrose for 2 min then in fresh fixative for 60 min. After washing samples in 0.1 M cacodylate with 5% sucrose buffer (pH 7.5), cells were sent to Otago Micro and Nanoscale Imagining (University of Otago, Dunedin, New Zealand) for secondary fixation in 1% osmium tetroxide in 0.1 M cacodylate followed by staining with 1% uranyl acetate in deionized water. Samples were then dehydrated and embedded in EmBed 812. Ultrathin sections were analysed on a Phillips CM100 Transmission Electron Microscope (Eindhoven, The Netherlands) fitted with a Megaview III camera (Olympus Soft Imaging Solutions GmbH, Munster, Germany).

### 2.8. ATP Analysis

Neutrophils (1 × 10^6^/mL) were stimulated with PMA (20 nM) at 37 °C in 5% CO_2_ in the presence or absence of DPI (10 µM). Samples were removed at intervals and assayed using a PerkinElmer ATPlight assay kit according to manufacturers’ instructions. Luminescence was measured using a POLARstar plate reader (BMG Labtech, Ortenberg, Germany).

### 2.9. Preparation of Neutrophil Lysates and Western Blotting for Histone Citrullination

Neutrophils (2.5 × 10^6^/mL) were incubated for 30 min in the presence or absence of Cl-amidine (200 µM) at 37 °C and then stimulated with ionomycin (3.5 µM). Cells were transferred to 12-well plates (1 ml/well) and incubated for 2 h at 37 °C in 5% CO_2_, collected on ice, transferred to 1.5 mL tubes and pelleted. Pellets were suspended in RIPA buffer [Tris-HCl pH 7.4 (50 mM), sodium deoxycholate (0.5%), sodium chloride (150 mM), SDS (0.1%), EDTA (1 mM) and Nonidet P-40 (1%)] containing protease inhibitor cocktail (1×) and phenylmethylsulfonyl fluoride (5 mM). Lysates were briefly boiled in reducing sample buffer containing 320 mM β-mercaptoethanol, resolved by SDS-PAGE and transferred to polyvinylidene difluoride membranes (Millipore). Samples were blocked with 5% milk in Tris-buffered saline with 0.05% Tween 20 (TBST) for 1 h, probed with anti-citH3 (1/2000 in 5% milk in TBST and detected with goat anti-rabbit horseradish peroxidase conjugate diluted 1/20,000. Bands were detected using an ECL Plus Western blotting detection system and visualised using the Q9 Advanced Chemidoc (UVItec, Cambridge, UK).

### 2.10. Live Cell Imaging

Neutrophils (1 × 10^6^/mL) were stimulated with PMA or *P. aeruginosa* PAO1 (MOI 10) in the presence or absence of DPI, seeded (100 µL) into clear 96-well plates and incubated at 37 °C in 5% CO_2_. At 30 and 45 min after stimulation, some cells were treated with DPI. Four hours after PMA addition, NETs were stained with Sytox green (200 nM). Cells were viewed on an Olympus IX-81 live cell inverted microscope and images captured using a XM10 monochrome fluorescence charge-coupled device camera and cell˄R software (Olympus Soft Imaging Solutions, Münster, Germany).

### 2.11. Phagocytosis Assay

Opsonized bacteria were added to warmed neutrophils (1 × 10^6^/mL) (MOI 10) and then separate 1.2 mL aliquots were seeded into 12-well plates and incubated at 37 °C in 5% CO_2_. At 30, 60 and 90 min, plates were transferred to ice and well contents transferred to tubes and cells pelleted. Supernatants containing non-ingested bacteria were collected along with two washings of the cell pellets, and dilutions were plated on sheep blood agar. Colonies were counted after overnight incubation at 37 °C.

### 2.12. Superoxide Production

Superoxide production was measured by following the reduction of cytochrome c (ε_550_ 21,100 M^−1^ cm^−1^). Briefly, neutrophils (5 × 10^5^) were pre-warmed at 37 °C in HBSS containing catalase (20 µg) and stimulated with PMA (20 nM). Samples were taken at intervals up to 2 h and added to cytochrome c (40 µM). The increase in A_550_ was measured over 5 min at each time point, and the rate of superoxide production was calculated from the steepest part of the slope. Specificity for superoxide was confirmed by stimulating cells in the presence of superoxide dismutase (20 µg).

### 2.13. Statistical Analysis

Statistical analyses were performed in GraphPad Prism version 8.1.2 (GraphPad Software, La Jolla, CA, USA). Differences between two groups were assessed by paired *t*-tests. Differences between greater than two groups were analysed by one-way ANOVA followed by Dunnett’s multiple comparisons tests. A *p*-value < 0.05 was considered significant.

## 3. Results

### 3.1. NET Formation with PMA, Pseudomonas aeruginosa (PAO1), Staphylococcus aureus and C. albicans Is Inhibited by the NADPH Oxidase Inhibitor DPI

**Figure 1:**
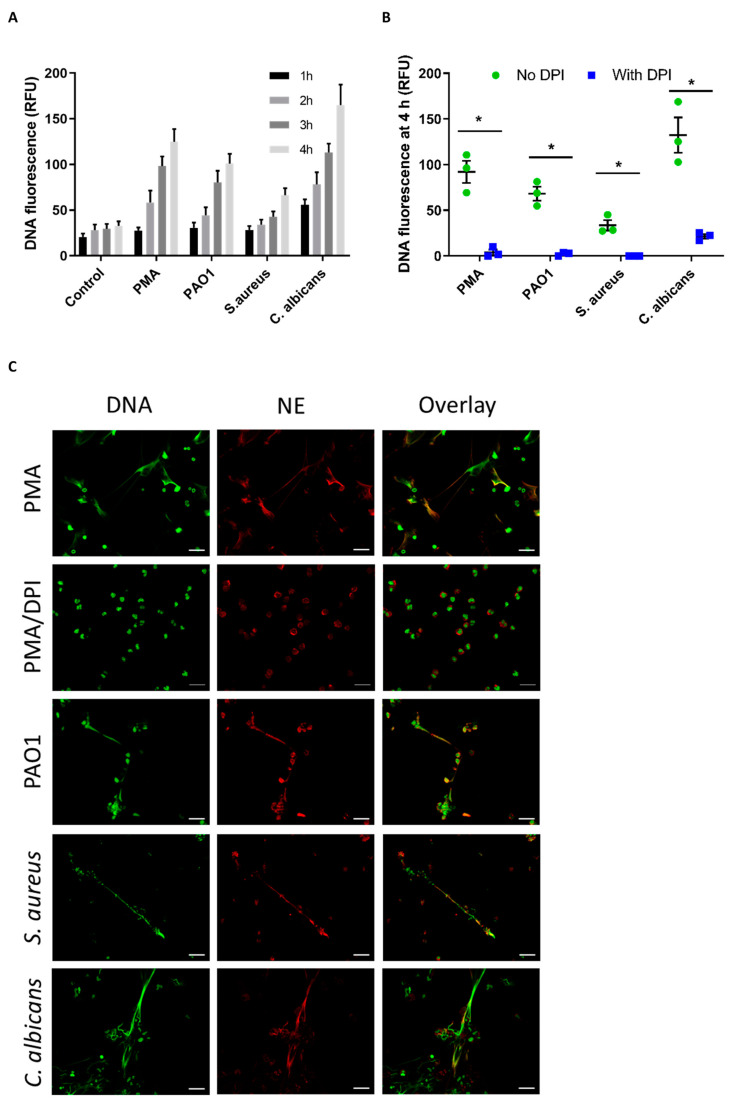
Time course and NADPH oxidase-dependence of NET formation with each stimulant. (**A**) Increase in DNA fluorescence over time when neutrophils were incubated either alone (Control) or with PMA (20 nM), *P. aeruginosa* PAO1 (MOI 10), *S. aureus* (MOI 10) or *C. albicans* (MOI 2). Data are means (SE) of three separate experiments. (**B**) Neutrophils were incubated with the same stimulants as in (**A**) in the presence or absence of the NADPH oxidase inhibitor DPI (10 µM) and DNA fluorescence was measured after 4 h. The fluorescence of control cells was subtracted from that of stimulated cells. Data are means (SE) of three separate experiments. *, significantly different than without DPI (*p* < 0.05 by paired *t*-test); (**C**) confirmation of NETs by immunofluorescence. Neutrophils were stimulated for 4 h in the presence or absence of DPI. Samples were then fixed and stained to show co-localization of DNA (green) with neutrophil elastase (NE) (red). Images are representative of at least three separate experiments. Scale bars represent 20 µm.

To monitor NET formation, we used the well-established plate assay that measures increase in fluorescence of the cell impermeable DNA dye Sytox green [25]. We observed a steady increase in DNA fluorescence over 2–4 h following stimulation of neutrophils with PMA or with opsonized *P. aeruginosa* PAO1, *S. aureus* and *C. albicans* (Figure 1A). NET formation induced by PMA and various microbial stimuli has been shown to require NADPH oxidase activity [8,9,10,11]. We confirmed this with the stimuli used in this study by showing that when NOX2 was inhibited by DPI, the increase in Sytox green fluoresescence was completely, or almost completely, suppressed (Figure 1B). To establish that the Sytox green fluorescence was due to NET DNA rather than simply an increase in permeability of the neutrophils, we also added each stimulus to the cells and, after 4 h, examined the fixed cells by fluorescence microscopy. Colocalization of DNA in strands with the NET marker neutrophil elastase (Figure 1C) confirmed that in each case NETs were formed in the absence but not the presence of DPI.

### 3.2. DPI Prevents Transition from Vacuolation to DNA Release

**Figure 2:**
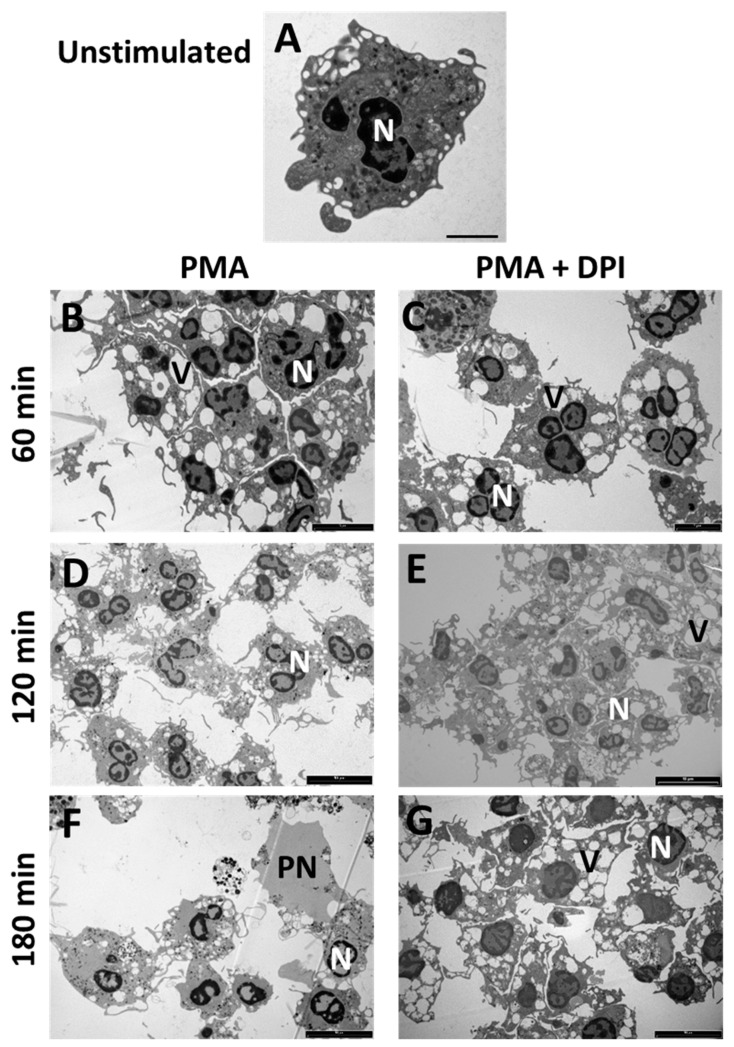
Transition electron micrographs showing morphology of PMA stimulated neutrophils in the presence or absence of DPI. (**A**) An unstimulated cell showing lobed nuclei and abundant cytoplasmic granules; (**B**–**G**) Neutrophils incubated with PMA for 60 to 180 min without (**B**,**D**,**F**) or with (**C**,**E**,**G**) DPI. Abbreviations: N = nucleus, V = vacuole, PN—pre-netting neutrophil. Results are representative of three separate experiments using different donors. Scale bars: (**A**) 2 µm; (**B**,**C**) 5 µm; (**D**–**G**) 10 µm.

**Figure 3:**
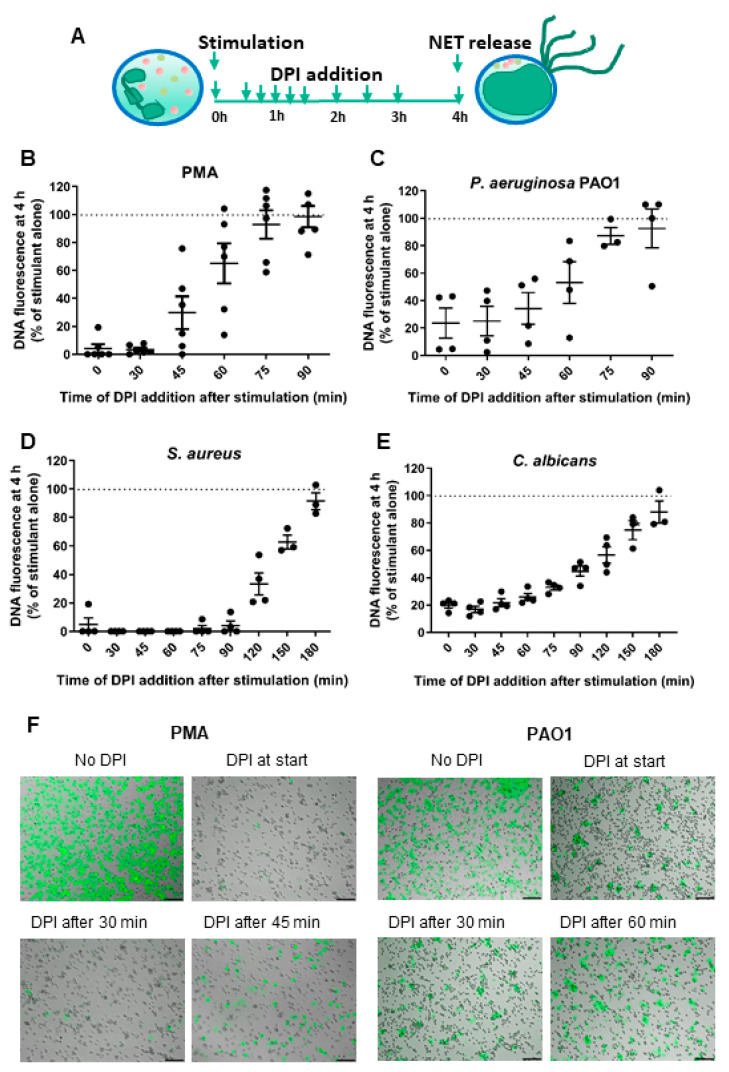
Reactive oxygen species produced by neutrophils early in the response to stimulation were not sufficient to induce NET formation. (**A**) Scheme of the experimental approach used to study the time when oxidants are important. Neutrophils were incubated with stimulant (time 0), and then DPI was added to inhibit further oxidant production at various time points after stimulation. After 4 h, the presence of NETs was assessed with Sytox green. (**B**–**E**) Neutrophils were stimulated for 4 h with PMA (20 nM), *P. aeruginosa* PAO1 (MOI 10), *S. aureus* (MOI 10) or *C. albicans* (MOI 2) in the presence or absence of DPI. Data are presented as the percentage of fluorescent DNA of cells with stimulant alone (No DPI). Data are means (SE) from 3–6 separate experiments with neutrophils from different donors. Stimulated cells treated with DPI were significantly different than stimulated without DPI (*p* < 0.05 by one-way ANOVA followed by Dunnett’s multiple comparisons tests) at the following times: PMA and *P. aeruginosa* PAO1 0–60 min; *S. aureus* and *C. albicans* 1–150 min. (**F**) Live cell microscopy of neutrophils incubated with PMA or *P. aeruginosa* PAO1 for 4 h in the presence or absence of DPI and stained with Sytox green. NETs appear as diffuse green fluorescence surrounding the cells. Live cell images were taken from six random fields of view per treatment and are representative of at least three independent experiments. Scale bars represent 100 µm.

Differences in the morphology of PMA-stimulated neutrophils in the presence and absence of DPI were examined by transmission electron microscopy. An unstimulated neutrophil with many cytoplasmic granules and a multi-lobed nucleus is shown in Figure 2A. Neutrophils stimulated with PMA developed numerous vacuoles within 60 min, whether or not oxidants were produced (Figure 2B,C). By 120 min, reduced vacuolation was observed in cells treated with PMA alone compared to those where oxidant production was inhibited (Figure 2D,E). At this time point, no obvious difference in nuclear morphology was apparent between the presence or absence of DPI. By 180 min, DNA expansion into the cytoplasm or release from the cell was evident with PMA alone (Figure 3F), but not when DPI was present (Figure 2G). From these observations, we conclude that DPI prevents the transition from vacuolation to DNA release into the cytoplasm that occurs between 2 and 3 h after stimulation. The persistence of vacuoles with DPI may at least in part be explained by their not being obscured by the nuclear expansion as occurs in stimulated cells without DPI.

### 3.3. Timing of the Requirement for Oxidant Production for NET Formation

To examine when neutrophil oxidants are required for NET formation, we incubated cells with PMA or microbial stimulants and then added DPI at various time points to prevent further NADPH oxidase activity. DPI stopped production of superoxide within a minute of addition (data not shown). Cells were then incubated for a total of 4 h and NET formation was assessed with Sytox green (see Figure 3A for experimental scheme). The addition of DPI up to 60 min after stimulation with PMA (Figure 3B) or *P. aeruginosa* PAO1 (Figure 3C), and up to 150 min after stimulation with *S. aureus* (Figure 3D) and *C. albicans* (Figure 3E) significantly inhibited NET formation. Live cell microscopy with PMA and *P. aeruginosa* PAO1 confirmed the decrease in fluorescence was due to a decrease in NETs (Figure 3F).

We examined if late inhibition by DPI could be related to prolonged phagocytosis, and associated NOX2 activity occurring over an extended period after the addition of bacteria. Rates of phagocytosis were assessed under NET-inducing conditions by measuring the decrease in extracellular bacteria over time. For *P. aeruginosa*, there was a 49 ± 1 (SD)% decrease at 30 min, and 65 ± 7% at 90 min; for *S. aureus*, the decrease was 52 ± 10% at 30 min, 49 ± 14% at 120 min). Microscopy showed that qualitatively there was a corresponding increase in neutrophil-associated bacteria, and, although not all were ingested, most were taken up by 90–120 min. Thus, NET formation could still be inhibited by DPI added after most of the phagocytosis had occurred.

**Figure 4:**
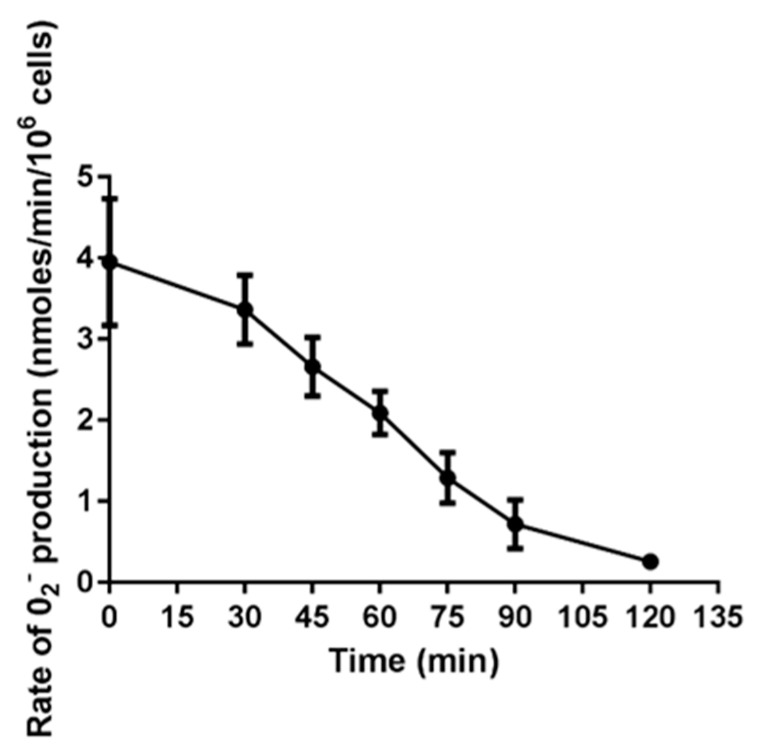
Rate of oxidant production in response to PMA. Neutrophils were stimulated with NET-inducing concentrations of PMA (20 nM) and the rate of superoxide production was monitored with cytochrome c. Measurements were for 5 min and rates taken from the steepest part of the slope. Data are means (SE) from 3–6 individual experiments.

To assess how oxidant production varied over the time until NET release, neutrophil superoxide production induced by PMA was measured. As expected, PMA the rate of superoxide production reached a maximum within 5 min, then gradually declined to half by 60 min, and near zero over 2 h (Figure 4). Thus, at 60 min, when the addition of DPI still inhibited NET formation, approximately 80% of the total superoxide had already been produced.

### 3.4. Alternative Effects of DPI

We chose DPI to study the timing of the NADPH oxidase activity requirement because it is a highly effective inhibitor at low concentrations, it acts quickly, and we are not aware of it acting on targets other than NOX2 in the neutrophil. However, DPI also inhibits other flavoenzymes, including those involved in mitochondrial bioenergetics. Even though the low concentration used here should be less effective at inhibiting non-heme flavoproteins than NOXs [26], we tested whether the suppression of NETs by DPI could have been due to inhibition of mitochondrial activity. However, under conditions where NET production induced by PMA gave 554 ± 71 RFU (SE; *n* = 3) in the Sytox green assay at 4 h and DPI decreased the signal to 2 ± 2 RFU, the complex I inhibitor, rotenone (at a non-toxic concentration; data not shown) had no effect (513 ± 67 RFU). As support for the DPI results, we considered using apocynin as another NOX inhibitor, but decided against this due to its complex mechanism that requires metabolism by MPO [27] and uncertainties about timing of the different steps. VAS2870, while not acting directly on the oxidase [28], is a possible option for further investigation.

**Figure 5:**
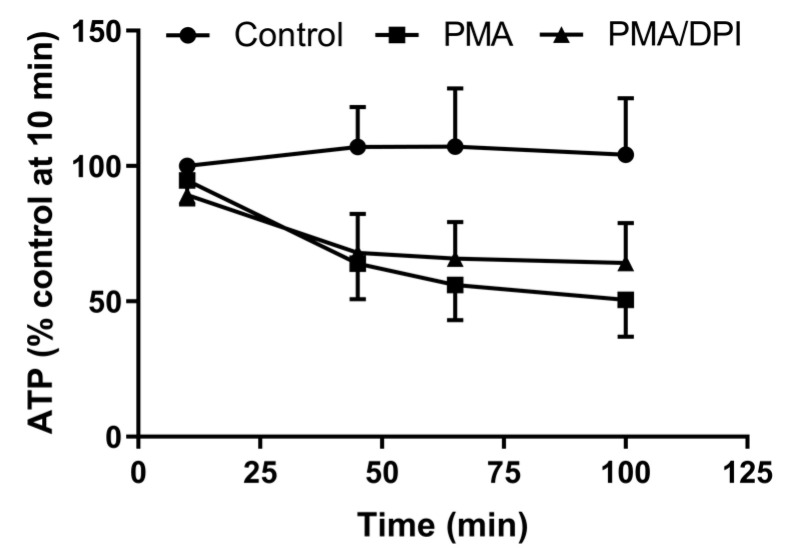
Effect of DPI on neutrophil ATP levels. ATP was measured at the indicated times in unstimulated (Control) neutrophils and cells stimulated with PMA in the presence or absence of DPI. PMA reduced cellular ATP in an oxidant-independent manner. Data are means and the range of two separate experiments.

As NET formation is an energy-requiring process [29], we examined whether inhibition by DPI could have been due to depletion of cellular ATP. However, this was not the case, as the decrease in ATP measured 45 min after PMA stimulation was the same in the absence and presence of DPI (Figure 5).

**Figure 6:**
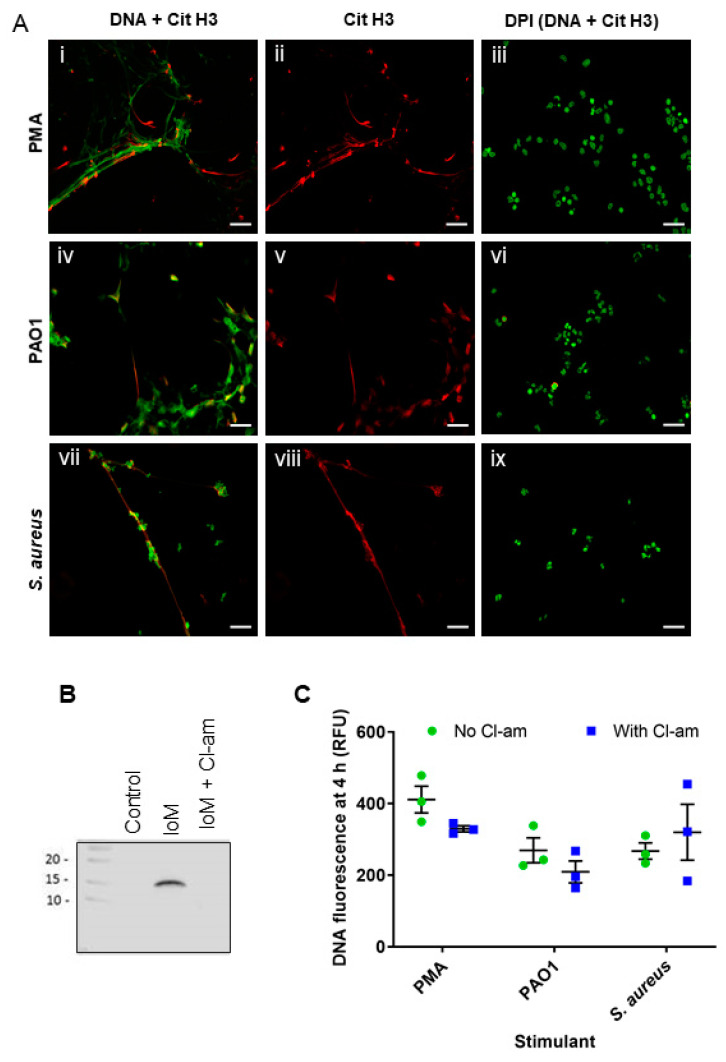
Oxidant-dependence of histone citrullination and effect of inhibition of PAD4 on NET formation. (**A**) Neutrophils were pretreated with DPI (**iii**,**vi**,**ix**) or media alone then incubated with PMA (20 nM) (**i**–**iii**) *P. aeruginosa* PAO1 (MOI 10) (**iv**–**vi**) or *S. aureus* (MOI 10) (**vii**–**ix**) for 4 h and then fixed and stained for NETs (green) and citrullinated histone H3 (red). DPI images show both green (DNA) and red (citH3) channels. Images are representative of at least three separate experiments. Scale bars represent 20 µm. (**B**) Western blot for citrullinated histone H3 (CitH3) showing that the PAD inhibitor Cl-amidine inhibits histone citrullination induced by ionomycin. Blot is a representative of two separate experiments. Control—unstimulated neutrophils; IoM—cells treated with ionomycin for 2 h; IoM + Cl-am—cells treated with ionomycin in the presence of Cl-amidine (200 µM). (**C**) Neutrophils were pretreated with Cl-amidine then incubated with PMA (20 nM), *P. aeruginosa* PAO1 (MOI 10) or *S. aureus* (MOI 10) for 4 h. DNA fluorescence was assessed by Sytox green staining. The fluorescence of control (unstimulated) cells was subtracted from that of stimulated cells. Data are means (SE) of three separate experiments.

Citrullination of histone H3 has been observed in association with NET formation with some stimuli [30,31]. We observed citrullination when NETs were induced with PMA (Figure 6A, panels i,ii), *P. aeruginosa* PAO1 (panels iv,v) and *S. aureus* (panels vii,viii). In each case, citrullination was blocked in the presence of DPI (panels iii,iv,ix). We examined the effect of blocking citrullination on NET formation using the pan-PAD inhibitor Cl-amidine, which we first validated by showing that it prevented citrullination of histone H3 in ionomycin-treated neutrophils (Figure 6B). Cl-amidine did not inhibit NET formation with any of the stimulants (Figure 6C) and on its own did not increase Sytox green fluorescence. Thus, histone citrullination was oxidant-dependent and accompanied NET formation but was not required for the process.

### 3.5. MPO Involvement in NET Production

**Figure 7:**
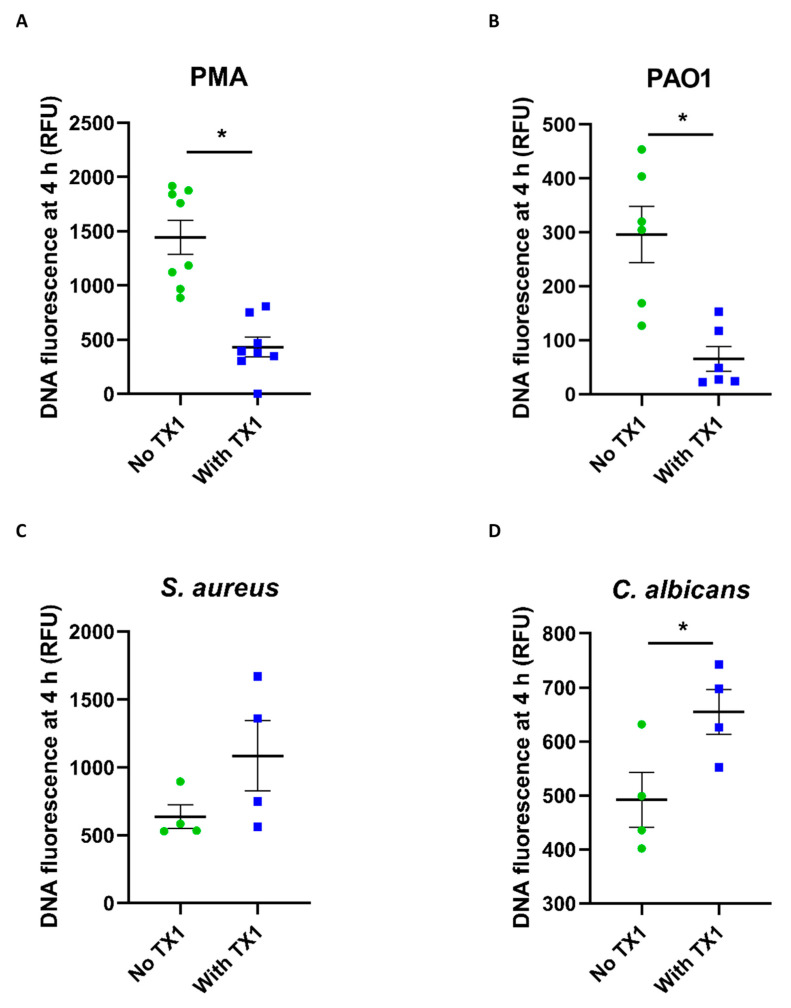
Effect of MPO inhibition on NET formation. Neutrophils were stimulated with (**A**) PMA (20 nM), (**B**) *P. aeruginosa* PAO1 (MOI 10), (**C**) *S. aureus* (MOI 10), or (**D**) *C. albicans* (MOI 2) in the presence or absence of the MPO inhibitor thioxanthine 1 (TX1) (10 µM), and DNA fluorescence was measured after 4 h. The fluorescence of control (unstimulated) cells was subtracted from that of stimulated cells. Data are means (SE) of 4–8 separate experiments. *, significantly different than without TX1 (*p* < 0.05 by paired *t*-test).

**Figure 8:**
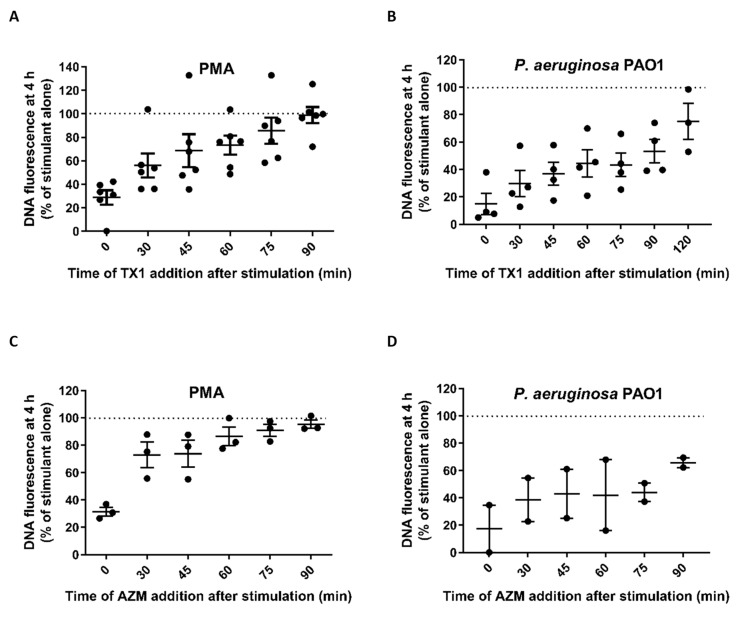
Early MPO activity is not sufficient to stimulate NET formation induced by PMA or *P. aeruginosa* PAO1. Neutrophils were stimulated with (**A**) PMA (20 nM) or (**B**) *P. aeruginosa* PAO1 (MOI 10). TX1 was added prior to or at the indicated times after stimulation. After 4 h, the presence of NETs was assessed by Sytox green plate assay. Data are presented as the percentage of fluorescent DNA of cells with stimulant alone (No TX1) and are means (SE) of 3–6 experiments with neutrophils from different donors. DNA fluorescence was significantly different than stimulated without TX1 (*p* < 0.01 by one-way ANOVA followed by Dunnett’s multiple comparisons tests) at the following time points: PMA 0–30 min; *P. aeruginosa* PAO1 0–90 min. (**C**,**D**) Neutrophils were stimulated under the same conditions with the MPO inhibitor AZM-198. Data are means (SE) of three independent experiments with PMA and means with range of two separate experiments with *P. aeruginosa* PAO1. DNA fluorescence was significantly different than stimulated without AZM-198 (*p* < 0.01 by one-way ANOVA followed by Dunnett’s multiple comparisons tests) only when AZM-198 was added prior to PMA.

Whether MPO participated in NET formation with the different stimuli was examined using the specific MPO inhibitor TX1 [23]. As shown in Figure 7, NET formation induced by PMA (A) and *P. aeruginosa* PAO1 (B) was significantly inhibited by TX1. In contrast, TX1 was not inhibitory with *S. aureus* (C) or *C. albicans* (D). We then investigated the timing of MPO inhibition with PMA and *P. aeruginosa* PAO1. NET formation was significantly inhibited when TX1 was added up to 30 min after stimulation with PMA (Figure 8A) and up to 90 min with *P. aeruginosa* PAO1 (Figure 8B).

We also examined the effect of another potent MPO inhibitor, AZM198, which was reported to be more effective than TX1 at blocking intracellular MPO activity and NET release induced by PMA [15]. We observed inhibition by AZM198 added at the start, but to a similar extent to that observed with TX1 (Figure 8C). The impact of delayed addition is consistent with the pattern observed with TX1. Our more limited investigation of AZ298 with *P. aeruginosa* PAO1 (Figure 8D) did not allow statistical analysis but is consistent with inhibition being similar to that of TX1.

### 3.6. Effect of Adding Hydrogen Peroxide after Inhibiting the NADPH Oxidase

**Figure 9:**
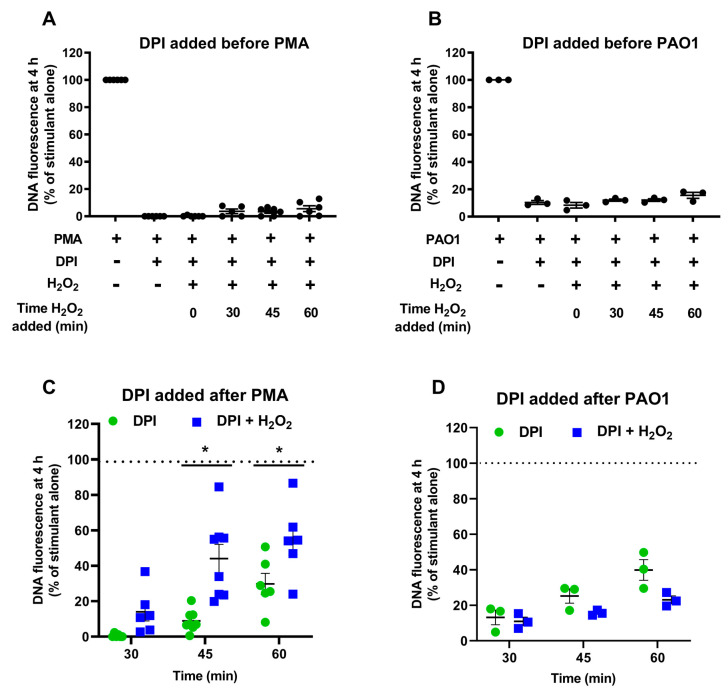
Effect of adding H_2_O_2_ to DPI-treated stimulated neutrophils. Neutrophils were stimulated for 4 h with (**A**,**C**) PMA (20 nM) or (**B**,**D**) *P. aeruginosa* PAO1 (MOI 10). DPI was added before stimulation (**A**,**B**) or at the indicated times after stimulation (**C**,**D**). A 100 µM bolus of H_2_O_2_ was added at the indicated times. Cells were then incubated for a total of 4 h and NET formation was assessed with Sytox green. Data are presented as the percentage of fluorescent DNA of cells with stimulant alone (No DPI). Data are the means (SE) of 3–6 separate experiments. *, significantly different to DPI alone at the same time point (*p* < 0.001 by paired *t*-test).

Having shown that a later oxidant-dependent step is required for NETs to form with PMA and the microbial stimuli, we reasoned that, if this alone were sufficient to induce NETs, exogenous oxidant addition at a later time might overcome inhibition of NOX2 activity. However, when neutrophils were stimulated with PMA or *P. aeruginosa* PAO1 with DPI present from the start, treatment after 30, 45 or 60 min with 100 µM H_2_O_2_ (Figure 9A,B) or 200 µM H_2_O_2_ (data not shown) did not facilitate the formation of NETs. Alternatively, if both early and late oxidants were required, allowing early NOX2 activity and adding H_2_O_2_ along with DPI at later times might enable NET formation. With PMA, co-addition of DPI and 100 µM H_2_O_2_ 30–60 min after stimulation did give a significant increase in NET formation compared with DPI alone (Figure 9C). Single doses of 50 or 200 µM, and repeated doses of 10 µM/min over 10 min, also had an effect but no more than that with the 100 µM bolus (data not shown). With *P. aeruginosa* PAO1, the addition of 100 µM H_2_O_2_ with DPI at any time had no restorative effect on NET formation (Figure 9D). Addition of H_2_O_2_ alone (100 or 200 µM) to neutrophils did not induce NET formation (data not shown).

## 4. Discussion

The main novel finding of our study is that a range of microbial stimuli induceoxidant-dependent NET release from neutrophils that can be suppressed by an inhibitor of NOX activity added well after addition of the stimulus. These results substantiate those reported for PMA [15] but, as has been stressed by others [1,22], before drawing mechanistic conclusions, it is vital to establish whether similar changes occur with more physiological stimuli. Our results demonstrate that the requirement for NOX2 activity at a late stage of NET formation is a more general phenomenon. This is surprising because the vast majority of oxidant production will have occurred prior to addition of the inhibitor. As shown here, the oxidase assembles within minutes of PMA addition, activity peaks early, and around 80% of the oxidant production occurs in the first hour. Likewise, with microbial stimuli, NOX2 activity commences soon after phagocytosis and tails off in each phagosome after about 15–20 min [12,32]. As DPI added after this time was still inhibitory, our results imply that NET production requires an oxidant step at a later stage when little oxidant production persists.

Regarding the role of MPO in NET formation, our results support a strong dependence on MPO activity when the stimulus is PMA, as demonstrated in other studies [6,10,15,17,33]. However, our finding that MPO inhibitors did not suppress NET formation with *S. aureus* and *C. albicans* indicate that MPO activity is not required with all stimuli. This is in agreement with previous findings with these organisms [10,13,34]. However, the situation is complex as we, and others [19], observed a dependence on MPO activity with *P. aeruginosa* PAO1. Intriguingly, this contrasts with a clinical isolate of *P. aeruginosa* we used previously, for which MPO inhibition had no significant effect [10]. Thus, while NOX activation and the production of superoxide/hydrogen peroxide appear necessary for NET production induced by microbial stimuli, only in some cases is the HOCl or other products generated by MPO required. What defines the different situations, and what specific oxidative reactions are involved in each case, remains to be established. Nevertheless, the similarity in timing profile for inhibition by DPI regardless of whether or not NET formation was dependent on MPO activity, plus the similar profiles for MPO inhibition and NOX2 inhibition when MPO was involved, suggest common step(s) regardless of the oxidant responsible.

Our DPI and MPO inhibitor findings indicate that a later oxidation step is necessary for NET formation but do not exclude the possibility that an earlier oxidation step is also needed. It is known from previous studies, carried out mainly with PMA, that the steps preceding NET release include intracellular reorganization, dissolution of the nuclear membrane and nuclear expansion, decondensation of chromatin and finally its expulsion from the cell along with various granule and cytoplasmic proteins, [1,3,5,8]. The rapid vacuolation we observed in response to PMA is well documented [35]. As it occurred in the presence of DPI, and is seen with CGD neutrophils [36,37], it is presumably not a part of the oxidative response required for NET formation.

One well documented early event is the release of active elastase from azurophil granules into the cytoplasm and, following dissolution of the nuclear membrane, uptake into the nucleus [17,18,33,38]. Maximal elastase release is seen within 1 h of PMA stimulation and translocation to the nucleus within 2 h [18]. Nuclear changes have also been detected within 30 min, and merging of elastase and histone staining within the nucleus by 2 h [8]. As these changes were not observed with CGD neutrophils or when MPO was inhibited, there is strong evidence that oxidant generation is required. However, the early timing of elastase activation, which coincides with the majority of oxidant production, does not fit with it being the step inhibited by late addition of DPI. Fewer details are known about phagocytic stimuli, but assuming they cause elastase mobilization over a similar time course to that with PMA, then it is likely that an early oxidative step is involved. A likely scenario, therefore, is that there is more than one oxidant-dependent step in NET formation, the first occurring early and related to elastase activation and an unidentified later step that requires only a low level of oxidant production. In attempting to address a two-step model, we did see partial restoration of NET formation with PMA when H_2_O_2_ was added together with DPI at later time points, but this was not the case with *P. aeruginosa*. There are limitations with this system as reagent H_2_O_2_ is less effective than endogenous generation at inducing NETs, and further exploration is required.

Later aspects of NET formation that could potentially be oxidant-dependent include dissolution of the nuclear membrane and expansion of the nucleus. As we observed by electron microscopy, this is inhibited by DPI, but it could also be downstream of initial oxidation. Another possibility is activation of PAD4 and histone citrullination [30,31]. This is a typical feature of NETs and, as observed here, dependent on NOX activity. Citrullination would facilitate chromatin decondensation and nuclear expansion, and has been widely considered as an essential step in many forms of NET formation. However, findings are equivocal [1,2,39,40], and our observed lack of inhibition by Cl-amidine is consistent with other evidence [6,41] that PAD4 activation accompanies but is not essential for NET formation. However, histone citrullination does appear to promote inflammatory responses to NETs once-formed [41]. Björnsdottir et al. [15], in their study of PMA stimulated NETs, propose that the late oxidant-dependent step involves NOX2/MPO activity that assembles at intracellular granule sites. However, the oxidative changes are still to be elucidated.

## 5. Conclusions

In conclusion, our study of oxidant-dependent formation of neutrophils NETs has highlighted a complex mechanism that can vary depending on the stimulus. MPO plays more of a role with some stimuli than others and with PMA and phagocytic stimuli, an oxidative step that is sensitive to inhibitors of NOX2 and in some cases MPO added well after the stimulus is important. This is most likely additional to the oxidant-dependent mobilization of elastase observed by others that occurs in the early stages after stimulation. More work is needed, however, to identify the specific oxidants and targets involved.

## Data Availability

The data presented in this study are available in this manuscript.
